# A Molecular Dynamics (MD) and Quantum Mechanics/Molecular Mechanics (QM/MM) Study on Ornithine Cyclodeaminase (OCD): A Tale of Two Iminiums

**DOI:** 10.3390/ijms131012994

**Published:** 2012-10-11

**Authors:** Bogdan F. Ion, Eric A. C. Bushnell, Phil De Luna, James W. Gauld

**Affiliations:** Department of Chemistry and Biochemistry, University of Windsor, Windsor, ON N9B 3P4, Canada; E-Mails: ionb@uwindsor.ca (B.F.I.); bushne1@uwindsor.ca (E.A.C.B.); deluna@uwindsor.ca (P.D.L.)

**Keywords:** OCD, L-proline, crystallin, oxidative deamination, iminium

## Abstract

Ornithine cyclodeaminase (OCD) is an NAD^+^-dependent deaminase that is found in bacterial species such as *Pseudomonas putida.* Importantly, it catalyzes the direct conversion of the amino acid L-ornithine to L-proline. Using molecular dynamics (MD) and a hybrid quantum mechanics/molecular mechanics (QM/MM) method in the ONIOM formalism, the catalytic mechanism of OCD has been examined. The rate limiting step is calculated to be the initial step in the overall mechanism: hydride transfer from the L-ornithine’s C_α_–H group to the NAD^+^ cofactor with concomitant formation of a C_α_=NH_2_^+^ Schiff base with a barrier of 90.6 kJ mol^−1^. Importantly, no water is observed within the active site during the MD simulations suitably positioned to hydrolyze the C_α_=NH_2_^+^ intermediate to form the corresponding carbonyl. Instead, the reaction proceeds via a non-hydrolytic mechanism involving direct nucleophilic attack of the δ-amine at the C_α_-position. This is then followed by cleavage and loss of the α-NH_2_ group to give the Δ^1^-pyrroline-2-carboxylate that is subsequently reduced to L-proline.

## 1. Introduction

l-proline (Pro) is one of the naturally occurring 20 genetically encoded amino acids and is unique amongst them in being the only one with a secondary α-amine. The effect of its structure and functionality upon the proteins in which it is found has long been studied. In addition, however, it is also known to have a number of key physiologically important roles. For example, it has been shown to be an important antioxidant needed by microorganisms, plants, and animals [1–8]. Furthermore, in certain pathogenic bacteria, it has been suggested that proline metabolism plays a role in enabling the pathogen to survive under harsh conditions [9–11]. Plants also depend on proline production for cell wall biosynthesis, while mediating abiotic and biotic cell stresses [12,13]. In mammals, the interconversion between proline and Δ^1^-pyrroline-5-carboxylate (P5C), an intermediate in its biosynthesis and catabolism, is believed to be involved in cell apoptosis [1,14–18]. Importantly, this cycle provides a redox shuttle between the cytosol and mitochondria, controlling the formation of reactive oxygen species [14,19,20]. Consequently, it is important to understand the functions of the enzymes that metabolize proline.

In general, Pro is synthesized within cells and organisms via multi-enzymatic pathways from either glutamate (Glu) or arginine (Arg) [9,14,21–24]. In the former, the enzymes involved require the use of γ-glutamyl kinase and glutamate-γ-semialdehyde dehydrogenase, which require the cofactors ATP and NADH or NADPH, respectively. In contrast, the latter pathway utilizes the metalloenzyme arginase and the pyridoxal-5′-phosphate (PLP)-dependent enzyme, ornithine δ-minotransferase. Both pathways, however, lead to the formation of a common intermediate, glutamic-semialdehyde. This then undergoes a non-enzymatic cyclization via an intramolecular condensation reaction to give the imine, P5C [14,25]. Then, the common enzyme P5C reductase (P5CR) reduces the latter cyclic intermediate to form L-Pro [9,26]. In mammals, the former pathway in which glutamate is converted to proline is believed to be the major metabolic route.

More recently, however, several bacteria have been shown to be able to utilize an unusual enzymatic route for proline biosynthesis [27,28]. More specifically, they use the NAD^+^-dependent non-metalloenzyme ornithine cyclodeaminase (OCD) to directly convert the amino acid L-ornithine (L-Orn), itself also an intermediate along the above “Arg-pathway”, to proline [9]. Thus, in contrast to the two general pathways discussed above in which the actual cyclization step is done without enzymatic participation, the mechanism of OCD includes the cyclization of a linear intermediate to a cyclic product (Scheme I) [9,29,30]. Furthermore, it not only produces L-proline stereospecifically and without additional enzymes being involved, but does so in an irreversible fashion [9,31].

It is generally believed that the overall mechanism of OCD begins with an initial hydride transfer from the L-Orn substrate’s C_α_–H moiety onto the C_4_ center of the nicotinamide ring of the NAD^+^ cofactor. This results in the formation of an iminium (C_α_=NH_2_^+^ containing) intermediate complex [32–37]. Unfortunately, however, it is then unclear how the mechanism proceeds. Based on the results of experimental studies including X-ray crystallographic structures and mass spectroscopic data, two possible pathways have been proposed (Scheme II). While they share some common features such as involving formation of a Schiff base, a chemically and biochemically important reaction process that has been previously studied in detail both experimentally and computationally, they also have some very important differences [38–47]. In particular, in the hydrolytic pathway, an active site water nucleophilically attacks the C_α_ center of the iminium intermediate, resulting in loss of ammonium with formation of the keto acid, 5-amino-2-oxopentanoate (Scheme IIA) [28,34]. This then undergoes a cyclization reaction in which the δ-amine nucleophilically attacks the carbonyl carbon to form a cyclic Δ^1^-pyrroline-2-carboxylate (P2C) species. This latter intermediate has previously been shown to be involved in other biochemical processes including, for example, lysine catabolism. It is further noted that this ring formation step can be considered analogous to the cyclization of glutamic γ-semialdehyde in which an amine (α-amine) reacts with a carbonyl carbon center. This step is then followed by a hydride transfer from the NADH moiety onto the C_α_-position (C2) of P2C to give L-proline.

In contrast, in the alternate proposed mechanism known as the non-hydrolytic pathway, and shown above in Scheme IIB [31], the δ-amino tail of the iminium intermediate is neutralized by a suitable active site residue. Consequently, it is now able to nucleophilically attack the C_α_-center of the iminium intermediate to form a cyclic 2-aminoproline species. This then undergoes loss of the α-amino group as ammonia or ammonium to give P2C. The final step is as in the hydrolytic pathway; hydride transfer from the NADH moiety onto the C_α_-position (C2) of P2C to give the final product.

As noted above, the enzyme OCD itself has only been found in a few select bacteria [27]. However, it has been noted that it shows close phylogenetic resemblance to some crystallin enzymes, in particular those found in mammals. These proteins and enzymes are primarily known for their role in lens and cornea structure and transparency [48,49]. However, their malfunctioning has been linked to a number of diseases including cataract formation and cancer. Indeed, OCD is a member of the μ-crystallin family of enzymes which have been found to be abundant in the eye lens of marsupials [50] and are believed to have a similar chemical mechanism to that of OCD [31,51]. Thus, a clearer elucidation of the mechanism of OCD can provide invaluable insights not only into its catalytic abilities and proline biosynthesis, but also into related physiologically important enzymes.

Computational chemistry is a proven invaluable tool for the study of enzymatic mechanisms [52]. Thus, we have complementarily applied molecular dynamics (MD) simulations and an ONIOM QM/MM approach to investigate the catalytic mechanism of OCD. In particular, we have examined the feasibility of both the proposed hydrolytic and non-hydrolytic pathways for conversion of L-ornithine to L-proline.

## 2. Computational Methods

### 2.1. Molecular Dynamics (MD) Equilibration

The Molecular Operating Environment (MOE) [53] program was used for model preparation and the molecular dynamics (MD) simulations. For the model, the structure of OCD used was taken from an X-ray crystallographic structure of an OCD homo-dimer; each active site was complexed with L-ornithine (L-Orn) and NADH (PDB ID: 1X7D) [31]. While the protein is homo-dimeric, we have chosen to use a single monomer for the MD simulations. This model simplification is reasonable since the catalytic site does not include interface residues. Thus, a single protomeric enzyme-substrate-cofactor complex was selected while the other was removed. In addition, all selenomethionine (Se-Met) residues were mutated to the native methionines. The bound NADH cofactor was oxidized to give the catalytically required NAD^+^ (*i.e.*, a hydride was removed from the C_4_ center of NADH). The coordinates of missing hydrogens were added using the MOE default method. A 7-Å spherical layer of water molecules was then added to solvate the enzyme-substrate complex. In order to force the system to lie within the volume of space defined by the surrounding shell of waters, an ellipsoidal potential wall with a scaling constant of 2 was placed around the solvated enzyme-substrate complex. To allow the electrostatic and van der Waals potentials to decay smoothly, a damping functional factor was included. The geometry of each solvated complex was then optimized using the CHARMM22 force field until the root mean square gradient of the total energy fell below 0.21 kJ mol^−1^ Å^−1^. The MD simulations were performed under constrained pressure and temperature. The equations of motion were coupled with the Nosé-Poincaré thermostat [54] and the time step for numerical integration was set to 2 fs. Initially, the system was heated from 150 to 300 K for a period of 50 ps, followed by an equilibration period of 100 ps at the constant temperature of 300 K and pressure of 1 atm. Based on rmsd and cluster analyses, a representative structure was chosen from the trajectory. This structure was then optimized with the CHARMM22 force field and used to obtain a suitable enzyme complex for further investigation (see below). We have successfully applied this MD protocol in the study of other enzymatic systems [55].

### 2.2. QM/MM Computations

The Gaussian 09 suite of programs [56] was used for all QM/MM calculations using the ONIOM formalism [57–65]. Optimized structures were obtained at the ONIOM (B3LYP/6-31G(d):AMBER96) level of theory [66–69]. Specifically, the AMBER96 molecular mechanics force field was used to describe the low-layer (*i.e.*, protein environment surrounding the active center) while the high-layer (*i.e.*, the active center) was described using the density functional theory-based method B3LYP in conjunction with the 6-31G(d) basis set. Harmonic vibrational frequencies were calculated at this same level of theory in order to obtain the corresponding Gibbs free energy corrections at SATP (ΔE_Gibbs_) in addition to characterize the nature of the stationary points on the potential energy surface (PES) (*i.e.*, as energy minima or first-order transition states (TSs)). Single point energy calculations on the above optimized structures were performed at the ONIOM (B3LYP/6-311 + G(2df,p):AMBER96//B3LYP/6-31G(d):AMBER96) + ΔE_Gibbs_ level of theory within a mechanical embedding (ME) formalism.

The final CHARMM22 optimized MD structure (see above) was used to obtain a suitable chemical model for the fully bound active site for use in the QM/MM-based mechanistic studies. More specifically, all residues and waters up to 15 Å from the L-ornithine substrate were extracted to be used as the enzyme-substrate-cofactor model. This distance was chosen as it has been previously shown that the steric and electrostatic effects arising from the protein environment surrounding the active site within this distance can have important effects on the mechanism and its intermediates and transition states [70].

**Figure 1:**
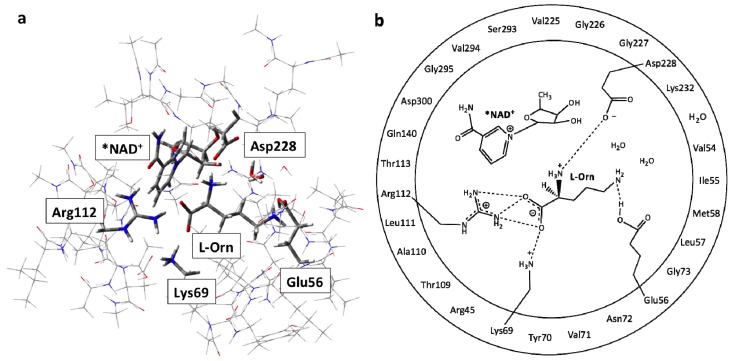
Illustration of the ONIOM (QM/MM) fully bound active site model used: (**a**) the low or molecular mechanics (MM)-layer is shown in wire while the high or quantum mechanics (QM)-layer is shown in tube format; (**b**) those moieties within the inner circle were included within the QM-region, while those within the outer circle represent the residues contained in the MM-layer of the QM/MM model.

The QM-region included the L-ornithine substrate and the active site groups with which it directly interacts or are experimentally known or proposed to be involved in the mechanism. Namely, the R-groups of Arg112, Lys69, Glu56 and Asp228 were included along with two active site waters observed within the MD simulations. The NAD^+^ cofactor was also included in part, the asterisk (*) indicating that only its nicotinamide and ribose ring was within the QM-region. The rest was replaced and modelled by a methyl group as shown in Figure 1. All those residues or waters shown in the outer-circle in Figure 1b were included within the MM-layer in their entirety. The only exceptions being those in which the R-group was included within the QM region. To ensure the integrity of the active site model during calculations, the α-carbons of each residue as well as that of the capping methyl in NAD^+^ were held fixed at their final CHARMM22 optimized positions (see above). This computational approach has been widely used previously, and successfully applied in studies on related enzymatic systems [55].

## 3. Results and Discussion

### 3.1. Structure of the Active Site with the Substrate L-Ornithine and NAD^+^ Cofactor Bound

**Figure 2:**
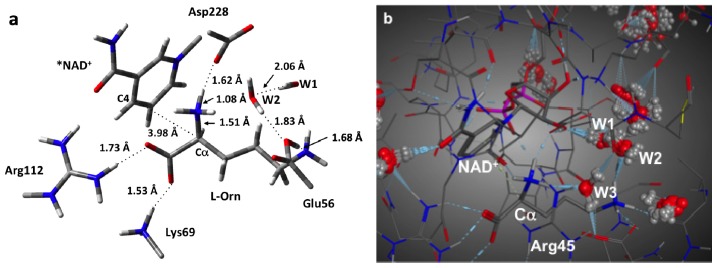
(**a**) Optimized structure (see Computational Methods) of the initial fully bound active site complex **RC** with selected bond lengths shown (in Angstroms). (**b**) Solvated MD structure of the active site.

We began this study by obtaining and examining the optimized structure of the fully bound active site complex (**RC**). That is, the complex in which both the NAD^+^ cofactor and L-ornithine (L-Orn) substrate are bound and which is shown in Figure 2a. As it can be seen, the L-Orn is bound electrostatically via interactions of each of its charged groups with active site residues. In particular, the α-COO^−^ forms short, strong hydrogen bonds with the protonated R-groups of both Arg112 and Lys69 with distances of 1.73 and 1.53 Å, respectively. Meanwhile, its α-NH_3_^+^ group is strongly hydrogen bonded with the nearby R-group carboxylate of the active site aspartyl (Asp228) with an *r*(α-NH···^−^OOC-Asp228) distance of 1.62 Å. Notably, this results in a modest lengthening in the participating N_α_–H bond to 1.08 Å. For the R-group, δ-NH_3_^+^, however, it was found that it transferred a proton to the R-group carboxylate of the active site glutamyl residue (Glu56) with which it hydrogen bonds. Although they now form a neutral N_δ_···HOOC–Glu56 hydrogen bond, its length is still decidedly short with a length of only 1.68 Å.

Importantly, in this bound conformation, the distance between the substrate’s C_α_ and the NAD^+^ cofactor’s C_4_ center to which a hydride would be transferred, *i.e*., *r*(C_α_···C_4_(NAD^+^)), is 3.98 Å. It is noted that while in the X-ray crystal structure (PDB ID: 1X7D), the enzyme was co-crystallized with the substrate and NADH, the above calculated distance is in reasonable agreement with the experimentally measured distance of 3.8 Å. It is noted that the mechanistically important C_4_···H–C_α_ distance is 3.51 Å and that the C_α_–H bond has lengthened marginally to 1.10 Å.

In addition, it is noted that in the MD structure of the initial reactant complex **RC** and shown in Figure 2b, three waters (W1, W2 and W3) were observed to lie in the active site and interact with the substrate. More specifically, one (W1) forms a hydrogen bonding bridge between the δ-NH_3_^+^ group and a sugar hydroxyl of the NAD^+^ cofactor. A second (W2) is simply hydrogen bonded to the substrates δ-NH_3_^+^ group while a third (W3) forms a hydrogen bonding bridge between the anionic R-group carboxylate of Glu56 and the substrate’s protonated α-NH_3_^+^ moiety. It should be noted that the oxygen of this latter water, O_W3_, is 3.50 Å away from the C_α_ center. These same interactions were also in agreement with the subsequent QM/MM optimized structure (not shown).

### 3.2. L-Orn Oxidation and Formation of 2-Aminoproline (AP)

**Figure 3:**
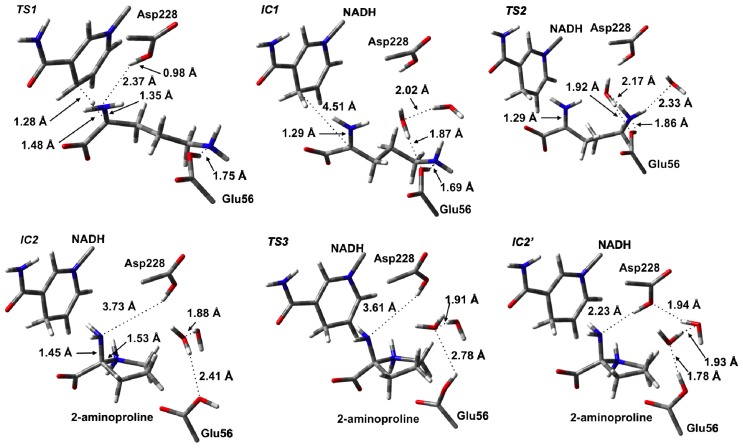
Optimized structures (see Computational Methods) with selected bond lengths shown (in Angstroms) for the ornithine cyclodeaminase (OCD) catalytic mechanism prior to deamination as L-ornithine is converted to 2-aminoproline.

**Figure 4:**
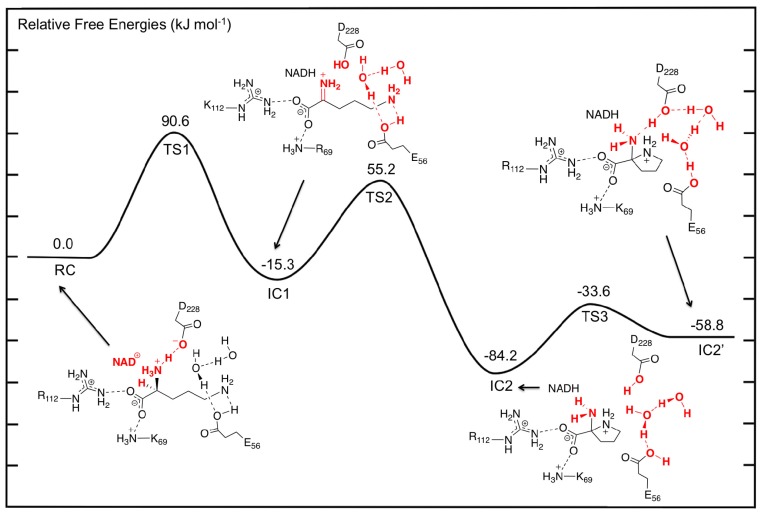
Potential energy surface (PES) obtained (see Computational Methods) of the OCD catalytic mechanism prior to deamination as L-ornithine is converted to 2-aminoproline.

The first catalytic step of OCD involves a hydride transfer from L-Orn to NAD^+^ via **TS1** (Figure 3). Specifically, the hydride is transferred from the C_α_–H moiety of the L-Orn substrate onto the C_4_ center of the nicotinamide ring of NAD^+^. This process occurs via **TS1** with a relatively high barrier of 90.6 kJ mol^−1^ with respect to **RC** (Figure 4). This may reflect in part the lack of any stabilizing interactions between the active site and the hydride entity as well as the large structural changes predicted. For example, as it can be seen in Figure 3, in **TS1** a significant reduction in the C_α_···C_4_(NAD^+^) distance is seen. Specifically, it has shortened by 1.24 Å to 2.74 Å. As a result, the hydride is almost equidistant between the C_α_ and C_4_ centers with C_α_···H^−^ and C_4_···H^−^ distances of 1.48 and 1.28 Å, respectively. However, concomitant with the H^−^ transfer, a proton from α-NH_3_^+^ group has transferred to the R-group carboxylate of Asp228 (Figure 3). Indeed, in **TS1**, the proton has fully transferred to the carboxylate as indicated by an *r*(H–O-Asp228) distance of 0.98 Å while the C_α_–N bond length has already significantly shortened by 0.16 Å to 1.35 Å. This suggests that the proton transfer occurs early in this reaction step.

Complete H^−^ transfer onto the NAD^+^ cofactor results in formation of the iminium ion intermediate (**IC1**) which lies lower in energy than **RC** by −15.3 kJ mol^−1^ (Figure 4). In **IC1**, the C_α_···C_4_(NADH) distance has lengthened significantly to 4.51 Å and is in fact now even greater than that seen in **RC** (Figure 3). Furthermore, the C_α_–N bond has shortened even further to just 1.29 Å indicating the formation of a Schiff base. In addition, the hydrogen bond interaction between the α-NH_2_ and now neutral Asp228-COOH group has been weakened with an *r*(Asp228-COOH···N_α_) distance of 4.14 Å.

As detailed in the introduction section, it has been proposed that subsequent to hydride transfer and formation of the Schiff base intermediate, cyclization of L-Orn may occur via one of two possible pathways. Namely, it may either involve a first reaction with an active water to give the keto acid, 5-amino-2-oxopentanoate (A2O), or direct nucleophilic attack of the δ-NH_2_ nitrogen at the C_α_ center. Indeed, the former A2O intermediate is similar to the glutamate γ-semialdehyde, common in both Argand Glu-pathways for proline biosynthesis. In particular, the ketone functionality is exposed to nucleophilic attack by either the α-amine in the non-enzymatic reaction or by δ-amine in OCD. As noted previously for **RC**, the nearest active site water that may nucleophilically attack the C_α_ center lies approximately 3.5 Å from the C_α_ center. We did attempt to elucidate a possible reaction pathway for reaction of **IC1** with a water molecule but were unable to do so. Analysis of the structure of **IC1**, however, suggests that in agreement with Goodman *et al.* [31], the steric presence of Arg45 packing against the substrate in **IC1** prevents the water from approaching much closer. Indeed, the nearest distance between Arg45 and the C_α_/N_α_ centers is only 3.40 Å. As a result, the hydrolytic pathway would seem unlikely to be physically feasible and hence is not being discussed further herein. Thus, the remainder of the discussion solely concerns the non-hydrolytic pathway unless otherwise mentioned. However, it is interesting to note that an iminium ion simulates a Lewis acid-activated carbonyl functional group [71]. In such Lewis acid activation, a lowering of the LUMO energy of the C=O π-system occurs promoting nucleophilic attack. Thus, it appears that OCD has optimized proline synthesis by maintaining the existence of a far more reactive intermediate such as an iminium ion rather than a ketone derivative.

With the iminium intermediate **IC1** formed, it is now susceptible to nucleophilic attack by N_δ_ of L-Orn. However, presumably prior to attack, the “tail” is required to curl within the active site. It is noted that the δ-NH_2_ group in **IC1**, unlike say the α-COO^−^ group, forms just one hydrogen bond interaction with an active site residue and that being with the now neutral R-group of Glu56. This would be expected to facilitate the required cleavage of this interaction and subsequent curling of the “tail” of **IC1**. Unfortunately, at the present level of theory we were unable to exactly optimize a TS for this process. Instead, a number of detailed systematic scans of the PES for such a curling was performed in order to determine an upper limit for the energy required for this step. In particular, it was found that a decrease in the ∠C_α_–C_β_–C_γ_–C_δ_ by 55.4° to 128.5° gave a structure (**TS2**) that corresponded to an energy maximum of 55.2 kJ mol^−1^ with respect to **RC**, which is 70.5 kJ mol^−1^ with respect to **IC1**. It is noted that in **TS2**, the N_δ_···C_α_ distance has decreased from 4.51 Å in **IC1** to 3.92 Å. Thus, clearly the desired C_α_–N_δ_ bond has not yet formed. Interestingly, it should be noted that all attempts to optimize energy minima complexes with ∠C_α_–C_β_–C_γ_–C_δ_ less than 128.5° (without constraining the C_α_–C_β_–C_γ_–C_δ_ dihedral angle) led directly to the formation of the very low energy 2-aminoproline intermediate complex **IC2** lying 84.2 kJ mol^−1^ lower in energy than **RC**. Importantly, in **IC2**, a C_α_–N_δ_ bond has been formed with a length of 1.45 Å, typical for a single C–N bond. This suggests that in OCD, the active site structure allows for a reasonably low energy curling of the “tail” of the Schiff base intermediate and that once suitably positioned, the N_δ_ center appears able to nucleophilically attack at the C_α_ center readily. Thus, as stated above, given that OCD has protected the iminium intermediate from reacting with water, it has allowed for an apparently barrierless C–N bond formation without the need for activation by a Lewis acid. This is far different than what has been seen in various carbonyl analogues where significant barriers for initial C–N bond formation exist [38–47].

### 3.3. Deamination of 2-Aminoproline (AP)

In order to lose the α-amine of **IC2** (*i.e.*, deamination), it must first be protonated. This is analogous to the required protonation of the hydroxide to form water prior to Schiff base formation in the aldehyde analogues [38,40,41]. One possibility is that an intramolecular proton transfer may occur from the ring –NH_2_^+^– group directly onto the leaving amine. However, this would necessarily involve a four-membered ring transition structure which is well-known to be higher in energy due to the inherent strain involved [72]. Alternatively, an active site residue may be able to act as a proton donor. Within the active site of OCD, however, the nearest acidic residue is the neutralized R-group carboxylic acid of Asp228. Within the present computational model in the formation of **IC2** (*i.e.*, during the preceding cyclization step), it was found that the Asp228-COOH group was no longer in a position to transfer a proton to the leaving α-amine (Figure 3). Thus, the Asp228-COOH···α-NH_2_ distance must first shorten allowing, for the formation of a hydrogen bond to the leaving α-amine. However, for this to occur **IC2** must undergo a conformational or hydrogen bonding network change. In particular, the proton on Glu56 must rotate allowing for a rearrangement of the active site waters allowing for the Asp228-COOH···α-NH_2_ distance to be reduced.

The process occurs via **TS3** with a barrier of 50.6 kJ mol^−1^ with respect to **IC2** to give the alternate complex **IC2**′ (Figure 4). The latter complex, while lying 25.4 kJ mol^−1^ higher in energy than **IC2**, still lies markedly lower in energy than RC by 58.8 kJ mol^−1^. As expected, this rotation of the proton on Glu56-COOH group caused Asp228-COOH to hydrogen bond with the α-amino group of the substrate. Furthermore, the hydrogen bonding network between the Glu56-COOH group and Asp228-COOH involving the two waters also changed. Specifically, the Glu56-COOH moiety now acts as a hydrogen bond donor via the waters to the carboxylic group of Asp228.

However, within the present computational model we were unable to locate a transition structure for proton transfer from Asp228-COOH onto the leaving amine. This is likely due to the fact that this would result in several charged groups (Asp228-COO^−^, α-NH_3_^+^ and –NH_2_^+−^), all being in close proximity and thus highly sensitive to the degree of inclusion of the electrostatics of the protein environment. While it is plausible that under experimental conditions Asp228-COOH may transfer its proton onto the α-NH_2_ group, previous computational investigations on Schiff base formation reactions have shown that such proton transfers generally occur via intramolecular proton transfer from the bridging amine; furthermore such proton transfers are facilitated by a water or other suitable group to prevent the formation of a four-membered TS [38,40,41].

**Figure 5:**
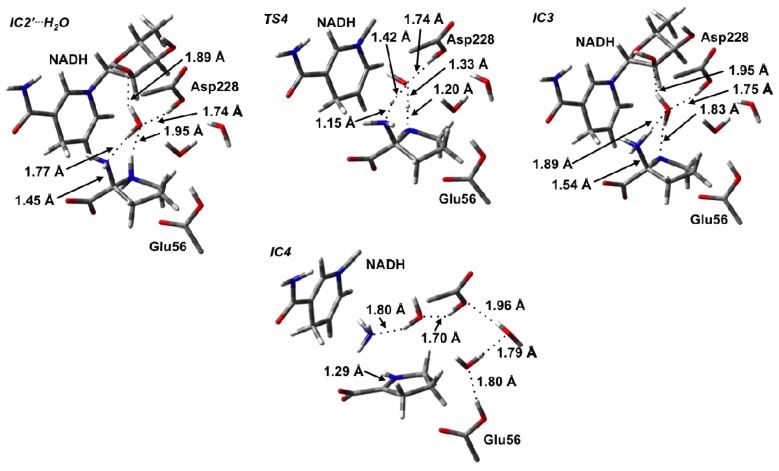
Optimized structures (see Computational Methods) with selected bond lengths (in Angstroms) of the water-assisted intermediate complex (**IC2**′···**H_2_O**), **TS4**, cyclic intermediate **IC3**, and Schiff base intermediate **IC4**.

**Figure 6:**
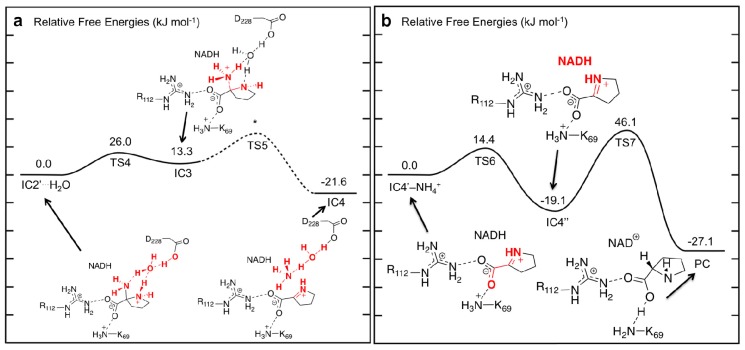
PESs obtained (see Computational Methods) for (**a**) water-assisted deamination of 2-aminoproline to give Δ^1^-pyrroline-2-carboxylate (P2C) and, (**b**) reduction of P2C to give L-proline.

Thus, we examined the possibility of a water molecule aiding proton transfer from either Asp228-COOH or the bridging –NH_2_^+^– to the α-NH_2_ moiety. Indeed, once the cyclic pyrrolidine species is formed in **IC2**′ it is noted that there is now solvent-accessible space in the vicinity of the Asp228-COOH group. The resulting **IC2**′ complex in which a water was added is hereafter denoted as **IC2**′···**H_2_O** (Figure 5). The resulting PES obtained for the subsequent proton transfers is shown in Figure 6a. Importantly, in **IC2**′···**H_2_O**, a water simultaneously acts as a hydrogen bond acceptor via its oxygen with the Asp228-COOH group and pyrrolidine’s –NH_2_^+^– moiety, while also acting as a hydrogen bond donor to the α-NH_2_ group of the pyrrolidine and the 2′-OH group of the NAD^+^ cofactor’s ribose. Indeed, in the optimized structure of **IC2**′···**H_2_O** *r*(Asp228-COOH···OH_2_) and *r*(αN···H_2_O) were found to be 1.74 and 1.77 Å, respectively, while for the ring amine *r*(NH_2_^+^···OH_2_) is slightly longer at 1.95 Å (Figure 5).

Interestingly, rather than a proton transfer from Aps228-COOH, it was found that the 2-aminoproline itself underwent an intramolecular proton transfer from its ring –NH_2_^+^– moiety onto the α-NH_2_ group via the bridging water molecule. This process occurred via the six-membered ring transition structure, **TS4**, at a very low cost of only 26.0 kJ mol^−1^ with respect to **IC2**′···**H_2_O** (Figure 6a). The resulting intermediate **IC3** formed lies only 13.3 kJ mol^−1^ higher in energy that **IC2**′···**H_2_O** (Figure 6a). Importantly, the protonation of the α-amine results in a significant lengthening in the C_α_–N_α_ bond in **IC3** by 0.09 Å to 1.54 Å and a shortening of the C_α_–N_δ_ bond (0.10 Å) within the pyrrolidine. Such bond changes are likely to aid in deamination and Schiff base formation.

In previous computational studies on Schiff base formation involving a ketone/aldehyde, the loss of the leaving group –OH_2_ has been shown to occur in two steps [38]. First the adjacent bridging amine undergoes an inversion thus allowing for favorable overlap of its lone pair and the anti-bonding orbital of C–OH_2_ bond to be cleaved. The second step is then cleavage of the C–OH_2_ bond itself. For the 2-amino-2-carboxy-pyrrolidine, no stable intermediate corresponding to inversion of the ring –NH– was obtained nor was a concerted TS involving cleavage of the C_α_–N bond with inversion of the bridging amine. However, it is noted that, while in previous studies, the barrier for loss of the water for the analogous carbonyl systems was found to be generally rate-limiting [38,39,41,42] cleavage of the C_α_–NH_3_^+^ bond in OCD was found to be exothermic. In particular, with **IC4** lying 34.9 kJ mol^−1^ lower in energy than **IC3** or −21.6 kJ mol^−1^ relative to **IC2**′···**H_2_O** (Figure 6a). Thus, there is clearly a driving force for formation of this Schiff base intermediate. C–N bond cleavage with concomitant Schiff base formation results in the Δ^1^-pyrroline-2-carboxylate (P2C) containing complex **IC4**. It should also be noted that in **IC4**, the Asp228-COOH proton has also transferred onto the leaving NH_3_ to give an ammonium ion, NH_4_^+^ via H_2_O. The latter is then free to leave the active site.

### 3.4. Hydride Transfer from NADH onto P2C to Give the Final Product L-Proline

**Figure 7:**
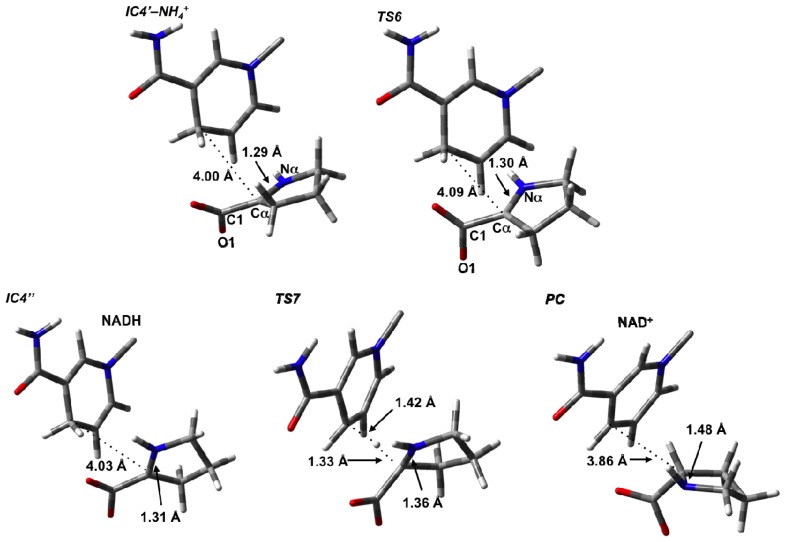
Optimized structures (see Computational Methods) with selected bond lengths shown (in Angstroms) of the Δ^1^-pyrroline-2-carboxylate intermediate **IC5**, **TS7**, and L-proline product (**PC**).

For the final stage, we considered formation of the L-proline product from P2C after loss of the cleaved NH_4_^+^ from the active site. Only minor structural changes were noticed in the resulting complex **IC4**′–**NH_4_** **^+^**. In principle, L-proline can be formed via hydride transfer from the NADH moiety on the C_2_ (what will become C_α_) of P2C. However, in **IC4**′–**NH_4_** **^+^**, the key _NADH_C_4_···C_2_ is quite long at 4.00 Å. Furthermore, the P2C itself is not ideally positioned for the transfer. However, it is able to undergo an intramolecular rotation about its C_2_–COO^−^ bond, *i.e.*, a change in its ∠O_1_–C_1_–C_α_–N_α_ dihedral angle, where the oxygen involved is the one hydrogen bonded to the Lys69. This process occurs via **TS6** at a markedly low cost of only 14.4 kJ mol^−1^ with respect to **IC4**′–**NH_4_** **^+^** suggesting that the rotation is likely reasonably unhindered (Figure 6b). It is noted that in **TS6** the ∠O_1_–C_1_–C_α_–N_α_ has decreased by 43.0° to 93.8°. This rotation is also exergonic with the resulting alternate conformer complex **IC4**″ being lower in energy than **IC4**′–**NH_4_** **^+^** by 19.1 kJ mol^−1^. Importantly, as a result of this reorientation of P2C within the active site, its C_2_ center is now more suitably positioned for hydride transfer and the _NADH_C_4_···C_2_ distance has decreased significantly to 3.14 Å (Figure 7).

Reduction of P2C via a hydride transfer from _NADH_C_4_–H onto its C_2_ center can then occur via **TS7** with a barrier of 65.2 kJ mol^−1^ with respect to **IC4**″ (Figure 6b). This is notably lower than the cost of the initial hydride transfer from the L-Orn substrate to NAD^+^. This final step is also exergonic with the final product complex (**PC**) in which the L-proline is bound within the active site being a further 8.0 kJ mol^−1^ lower in energy than **IC4**″, and with a relative free energy of −27.1 kJ mol^−1^ with regards to **IC4**′–**NH_4_** **^+^** (Figure 6b).

## 4. Conclusions

Using a combination of MD and QM/MM methods the catalytic mechanism of OCD has been investigated. From the results it was found that the initial hydride transfer from the C_α_–H group of the L-ornithine substrate to the C_4_ center of the NAD^+^ cofactor with concomitant formation of a Schiff base, is the rate limiting step. In particular, this process occurred with a relative free energy barrier of 90.6 kJ mol^−1^. Experimentally, this hydride transfer step might be examined by deuterating H–(C_α_) of L-Orn and conducting a kinetic isotope effect study.

For the remaining steps of the mechanism, while two pathways have been proposed, it appears that the enzyme most likely operates via a non-hydrolytic pathway. In particular, the MD and QM/MM results suggest that water is sterically hindered from attacking C_α_ after the initial Schiff base formation. Given that iminium ions are generally more reactive it seems that the active site of OCD evolved to guarantee such an intermediate exists by preventing its reaction with water. Indeed, following a conformational change of the substrate within the active site, a barrierless C–N bond formation occurred. This is considerably different than seen in previous investigations of various carbonyl analogues where significant barriers to C–N bond formation exists. While a transition state for deamination could not be found, the overall process was found to be thermodynamically favorable. Importantly, with deamination, a second Schiff base was formed. Like the initial C–N bond formation, this Schiff base would likely become more reactive. Indeed, the final step in the reaction was a H^−^ transfer with a low barrier of 65.2 kJ mol^−1^. The resulting product, L-proline, being thermodynamically more favored than the preceding intermediate.

Thus, while the typical biosynthetic pathway for L-proline from arginine requires two enzymes and non-enzymatic cyclization of a glutamate γ-semialdehyde intermediate, this cyclic intermediate being common to the pathway involving glutamate, is later reduced to form L-Pro. In contrast, ornithine cyclodeaminase appears to first exploit the formation of a highly reactive C_α_=NH_2_^+^–containing iminium ion in order to enable cyclization and ultimately formation of a second iminium ion, Δ^1^-pyrroline-2-carboxylate, formed after loss of the α-NH_3_ group. The latter ion is readily reduced via a hydride transfer from the NADH cofactor onto its “C_α_-center”, resulting in the formation of L-proline.

## Figures and Tables

**Scheme I:**
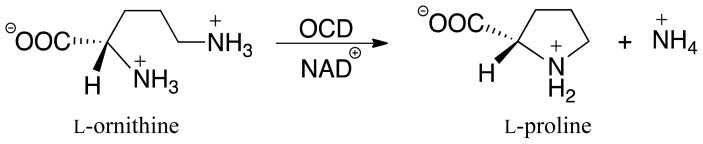
Overall reaction for conversion of L-ornithine to L-proline as catalyzed by ornithine cyclodeaminase (OCD).

**Scheme II:**
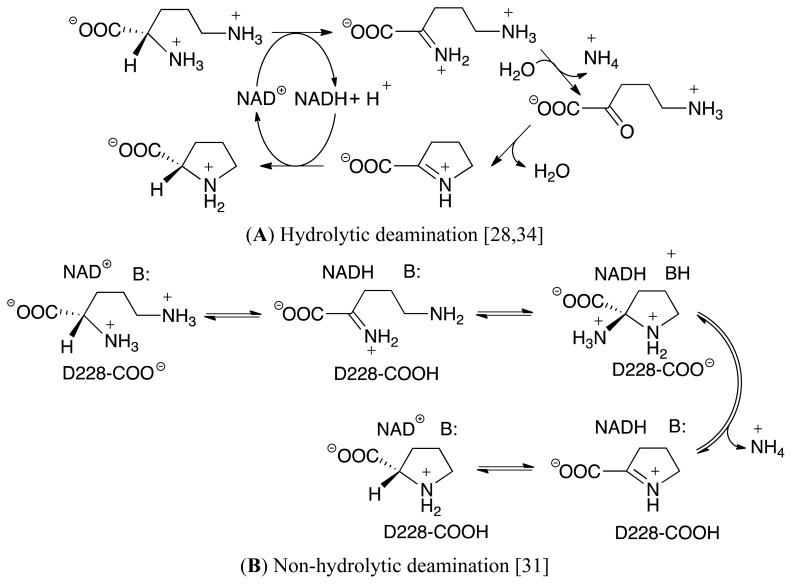
Proposed (**A**) hydrolytic and (**B**) non-hydrolytic pathways for the conversion of L-ornithine to L-proline as catalyzed by the enzyme ornithine cyclodeaminase.
